# Algorithm for pixel-level concrete pavement crack segmentation based on an improved U-Net model

**DOI:** 10.1038/s41598-025-91352-x

**Published:** 2025-02-24

**Authors:** Zixuan Zhang, Yike He, Di Hu, Qiang Jin, Manxu Zhou, Zongwei Liu, Hongli Chen, He Wang, Xinchen Xiang

**Affiliations:** 1 College of Hydraulic and Civil Engineering, Xinjiang Agricultural University, Ürümqi, 830052 China; 2 Xinjiang BIM and Prefabricated Engineering Technology Research Center, Ürümqi, 830052 China

**Keywords:** Concrete cracks, Semantic segmentation, Convolutional neural networks, U-Net, Deep learning, Civil engineering, Computer science

## Abstract

Cracks that occur in concrete surfaces are numerous and diverse, and different cracks will affect road safety in different degrees. Accurately identifying pavement cracks is crucial for assessing road conditions and formulating maintenance strategies. This study improves the original U-shaped convolutional network (U-Net) model through the introduction of two innovations, thereby modifying its structure, reducing the number of parameters, enhancing its ability to distinguish between background and cracks, and improving its speed and accuracy in crack detection tasks. Additionally, datasets with different exposure levels and noise conditions are used to train the network, broadening its predictive ability. A custom dataset of 960 road crack images was added to the public dataset to train and evaluate the model. The test results demonstrate that the proposed U-Net-FML model achieves high accuracy and detection speed in complex environments, with MIoU, F_1_ score, precision, and recall values of 76.4%, 74.2%, 84.2%, and 66.4%, respectively, significantly surpassing those of the other models. Among the seven comparison models, U-Net-FML has the strongest overall performance, highlighting its engineering value for precise detection and efficient analysis of cracks.

## Introduction

Cracks are a common form of pavement distress and indicator of pavement damage, and they have become a significant focus in the evaluation and repair of concrete pavement^1,2^. To prevent the deterioration of concrete pavements and ensure proper maintenance, it is essential to detect surface cracks accurately and assess their severity, providing data to support subsequent repairs^3,4^. Manual inspection is a widely used method for crack detection; however, this method is often inefficient and prone to inaccuracies^5^. Owing to factors such as the inspector’s expertise and subjective influences, the crack assessment process may consume a significant amount of labor and resources, and the results may vary considerably from the actual situation^6^. Additionally, relying solely on manual inspection for crack detection requires a considerable amount of labor and time, making it difficult to meet the demands for efficiency, accuracy, and continuity in recognition tasks^7,8^. Over the past few decades, scholars both domestically and internationally have researched numerous automatic crack detection methods^9,10^. These methods can be broadly classified into traditional image processing techniques (IPTs) and deep learning approaches that utilize convolutional neural networks^11^. Challenges faced when using traditional IPTs include complex backgrounds and multiscale features in images of concrete damage^12^. Therefore, there is a need to develop an efficient and accurate method for concrete crack detection^13^.

In the early stages of using digital IPTs to detect cracks in various structures, most methods were combinations or enhancements of traditional IPTs^14^, such as thresholding, edge detection, filtering, image synthesis, and segmentation^15–17^. Luxmoore et al.^18^ were among the first to discuss the application of holography in the non-destructive testing of concrete. In addition to its application scope, Hutchinson et al.^19^ proposed a statistical-based method grounded in Bayesian decision theory for extracting cracks in concrete structure images, offering a reliable and robust approach for analyzing large volumes of image data. Li et al.^20^ introduced a long-distance image acquisition device and an integrated image processing method featuring an improved image segmentation algorithm based on the C-V model, which enhances the accuracy and efficiency of crack detection. Although these crack detection methods based on traditional digital IPTs significantly reduce detection time and improve accuracy, they often rely on the assumption that crack pixels are darker than the background and are typically continuous. This assumption makes it challenging to apply these methods effectively in environments with complex background noise^21,22^.

With the rapid development of computer science and the exponential growth of computing power, deep learning has demonstrated high precision and efficiency in the field of image processing. Compared with traditional IPTs, vision-based deep learning methods feature more layers and parameters and are therefore better suited for complex scenarios with varying lighting conditions or high noise levels. Additionally, deep learning models can automatically learn features from large datasets. These methods are primarily categorized into three types—image classification, object detection, and image segmentation—all of which have been explored in the context of crack detection research^23^.

Classification-based methods focus primarily on determining whether road images contain cracks and identifying the types of cracks. Several typical convolutional neural network (CNN) architectures, such as ResNet, VGGNet, and DenseNet, have been widely adopted and have shown superior performance compared with traditional image processing algorithms^11^. Krizhevsky et al.^24^ trained a large deep CNN on a large database and model, utilizing data augmentation and dropout techniques to prevent overfitting; however, these techniques resulted in extended training times. Silva et al.^25^ developed a deep learning-based model using CNNs for detecting concrete cracks, achieving an accuracy of 92.27%, particularly when integrated with unmanned aerial vehicles (UAVs). While their method demonstrates high accuracy, there remains room for improvement in terms of efficiency, computational complexity, and handling larger, more complex datasets under varying environmental conditions, such as different lighting, weather, and road types, which are essential for real-world applications. Li et al.^26^ used the gradient-weighted class activation mapping (GradCAM) method to classify and weakly supervise the localization of cracks in the foundation of an arch dam without the need for manual annotations, achieving real-time detection and localization of defects in concrete dam structures. However, this classification model can only provide a rough location of the cracks and cannot accurately pinpoint them.

Object detection involves locating objects within an image using bounding boxes and determining each object’s category, addressing the challenge of accurately pinpointing the locations of cracks in images. Existing object detection methods, such as You Only Look Once (YOLO) and Single Shot MultiBox Detector (SSD), have been widely employed for crack detection^27^. Redmon et al.^28^ introduced the first version of YOLO, YOLOv1, in 2015, which brought new concepts and techniques to the field of object detection. In 2016, Liu et al.^29^ proposed SSD, a method that uses a single deep neural network for object detection. The SSD model is easy to train and can be directly integrated into systems requiring component detection. Subsequent versions of YOLO have built on this foundation, continuously improving detection accuracy and performance. For example, Liang et al.^30^ enhanced YOLOv5 by adding three modules, which significantly improved both average precision and inference speed. However, while these methods can identify the type and location of cracks, they do not provide high-precision information about crack size, such as length and area.

Segmentation-based methods identify target objects at the pixel level, where each pixel is classified as either a crack or non-crack pixel. This approach allows for the acquisition of pixel-level location information, enabling the extraction of more significant crack feature information from the detection results. Pixel segmentation can be divided into instance segmentation and semantic segmentation. One instance segmentation method was proposed by Zhang et al.^31^, who employed a deep learning approach based on a weakly supervised instance segmentation (WSIS) framework to detect cracks, introducing a novel dynamic balanced binary cross-entropy loss function. Fan et al.^32^ developed an ensemble of deep convolutional neural networks (without pooling layers) based on probabilistic fusion, which effectively measures crack length and width. Guo et al.^33^ proposed a real-time pixel-level detection framework that enhances real-time instance segmentation models by combining rapid object detection with highly accurate instance segmentation. The proposed model implements a backbone network with finer granularity and a receptive field. Ye et al.^34^ proposed an enhanced YOLOv7 network design that addresses challenges related to feature loss and the detection of small recognition frames and gradients, thereby increasing the model’s detection accuracy. However, crack detection methods based on instance segmentation have limitations. For example, these methods must differentiate between individual instances within the same category, increasing the relative complexity of the model. In scenarios involving large-scale image data or when real-time performance is critical, semantic segmentation is more advantageous. One semantic segmentation was proposed by Long et al.^35^, who developed the fully convolutional network (FCN), which can process inputs of any size and produce outputs of corresponding dimensions through efficient inference and learning. The FCN represents a foundational advancement in semantic segmentation. Many researchers subsequently proposed various segmentation networks, such as U-Net, SegNet and DeepLab, and applied these networks to crack segmentation^36^. Chen et al.^37^ proposed a deep learning-based semantic segmentation model that leverages advanced deep learning techniques to enhance the segmentation process, thereby facilitating more accurate and efficient detection. Zhang et al.^38^ proposed an FCN for concrete crack detection that leverages dilated convolution. This method incorporates dilated convolutions with varying dilation rates and a multibranch fusion strategy, achieving improved crack detection. Li et al.^39^ developed the multi-frequency network architecture, OUR-Net, which incorporates Octave Max Unpooling and Octave Convolution Residual Blocks to enhance pavement crack segmentation accuracy. Chu et al.^40^ utilized multi-scale feature fusion and an attention mechanism to enhance the accuracy of small crack detection in complex backgrounds. Shim et al.^41^ discussed the use of a multiscale and adversarial learning-based semi-supervised method for detecting cracks in concrete structures. This approach involves segmenting the image into regions that represent cracks or non-cracks, thereby improving the accuracy of detection. Additionally, semantic segmentation outperforms instance segmentation in terms of data annotation costs, overall scene understanding, and scalability.

Significant progress has been made in the field of crack detection, with important contributions from researchers worldwide. Matarneh et al.^42^ introduced a novel approach combining the DenseNet201 model and the Gray Wolf Optimizer (GWO) for asphalt pavement crack classification, achieving promising results and demonstrating high feasibility for practical applications. Wen et al.^43^ proposed a multi-scale context feature and cross-attention method based on convolutional neural networks, which effectively addressed segmentation challenges caused by blurry crack edges. Zhu et al.^44^ developed an efficient and accurate automated crack detection network, enhancing detection speed and efficiency through network lightweighting and depth-wise separable convolutions. Yao et al.^45^ proposed a new Pyramid Region Attention Module (PRAM) that combines pyramid pooling and optimized non-local (NL) mechanisms, enabling global multi-scale context integration and long-range dependency capture at relatively low computational cost, thus allowing for automatic, fast, and high-precision crack identification. Overall, researchers have significantly improved the accuracy and efficiency of pavement crack detection by integrating advanced deep learning models, such as YOLOv5 and CrackNet, along with innovative enhancements, driving the development of automated road monitoring and maintenance technologies.

U-Net, a CNN architecture introduced by Ronneberger et al.^46^ in 2015, is a notable example of semantic segmentation algorithms. Cheng et al.^47^ were among the first to apply U-Net to crack images, processing the images holistically to directly generate crack segmentation results. C Chen and He^48^ proposed a U-shaped encoder-decoder network combined with an attention mechanism for pixel-level detection and evaluation of road cracks. This approach significantly improves the detection of small cracks. Ji et al.^49^ proposed an automated crack detection method based on an advanced U-Net architecture that optimizes the retention of spatial information by utilizing U-Net’s encoder-decoder structure and skip connections, improving the accuracy of crack detection, especially in complex backgrounds. However, U-Net-based crack detection methods have limitations, such as increased computational complexity of the segmentation network, higher costs for pixel-level crack annotation, and requirements for detection accuracy and speed. In addition, U-Net-based methods may not fully detect extremely small cracks.

This paper proposes a new pixel-level semantic segmentation network called U-Net-feature map-multipath propagation-layer fusion (U-Net-FML). This network incorporates two innovations and a structural modification to extract sufficient crack detail information, achieving high accuracy, recognition rates, and crack detection speed even in complex environments. This model is specifically designed for precise crack detection and rapid crack analysis.

The primary contributions of this work can be summarized as follows:

1. The convolutional section is optimized to reduce parameters and model weight effectively.
2. A novel detection model employing block partitioning and multipath propagation improves accuracy for small cracks and those in complex backgrounds.
3. Multiscale feature integration, combined with pixel-level enhancement and smoothing techniques, boosts the model’s representational capacity and ability to capture detailed crack features.

## Proposed method

**Fig. 1:**
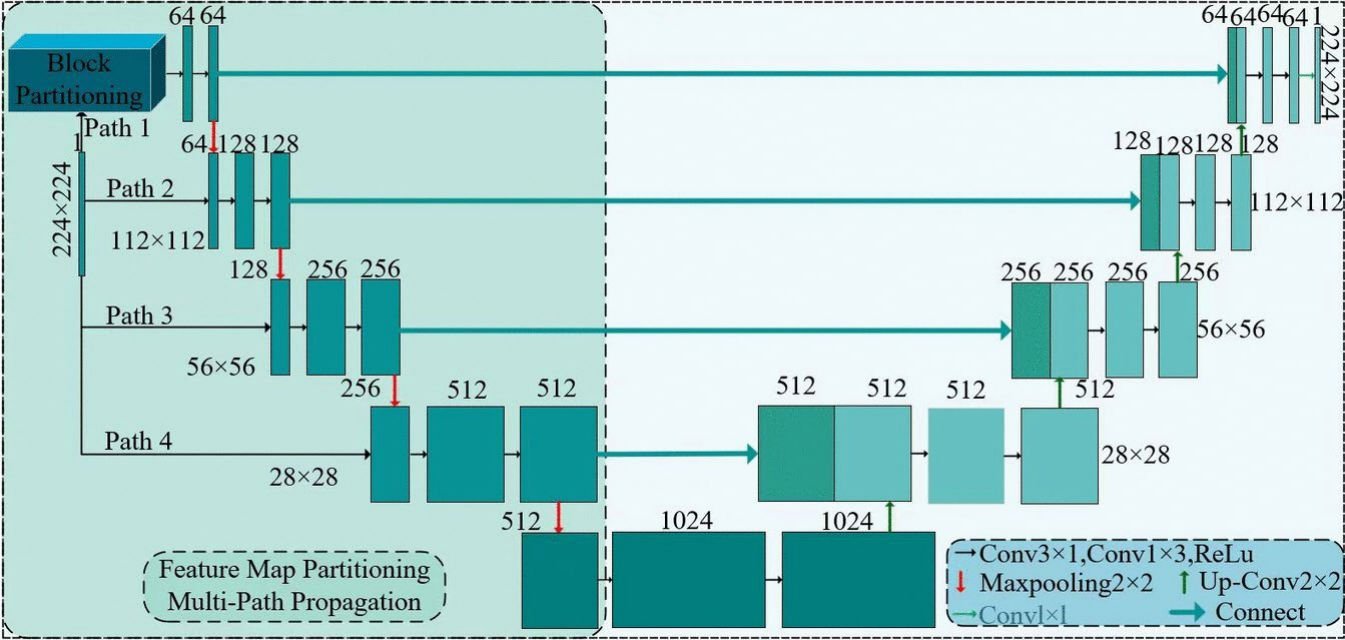
U-Net-FML network.

This paper proposes a U-Net-based network model for detecting pavement cracks, named U-Net-FML, as shown in Fig. 1. The left side of the figure represents the encoding process, consisting of four downsampling blocks, through which features are extracted from the input image and progressively compressed. The right side of the figure represents the decoding process, consisting of four upsampling blocks, which gradually restores the spatial resolution of the image. Each row in Fig. 1 corresponds to a different spatial size. Each box with solid borders in the figure represents a multichannel feature map, with the number of channels indicated at the top of the box. Additionally, as shown in the legend in Fig. 1, the arrows represent various operations. The fixed input size of the 2D images used in this study is 224 × 224 × 1, and the output segmentation map has the same dimensions.

U-Net has a typical encoder-decoder structure that gradually restores the spatial resolution of the image through deconvolution (transposed convolution) or upsampling operations, rebuilding low-resolution feature maps at a size close to that of the original image. Moreover, U-Net features skip connections, which directly connect feature maps from different stages of the encoder to their corresponding stages in the decoder. The fusion of low-level details and high-level semantic information retains the spatial information of the image and improves segmentation accuracy, particularly for boundaries and small targets^50,51^. Although U-Net performs well in semantic segmentation tasks, it also has several limitations. The network’s multiple convolutional layers and skip connections result in a high parameter count, which can slow computation, especially when handling high-resolution input images. Additionally, U-Net tends to blur complex edges, complicating the clear segmentation of fine structures, such as cracks, delicate details, or boundaries, often resulting in a loss of detail. To address the limitations of the original U-Net model, this study optimizes convolution operations to increase the training speed. Additionally, techniques such as feature map partitioning, multipath propagation, and multiscale feature fusion are employed to improve the model’s ability to distinguish small cracks from complex backgrounds, resulting in greater segmentation accuracy.

### Lightweight model

**Fig. 2:**
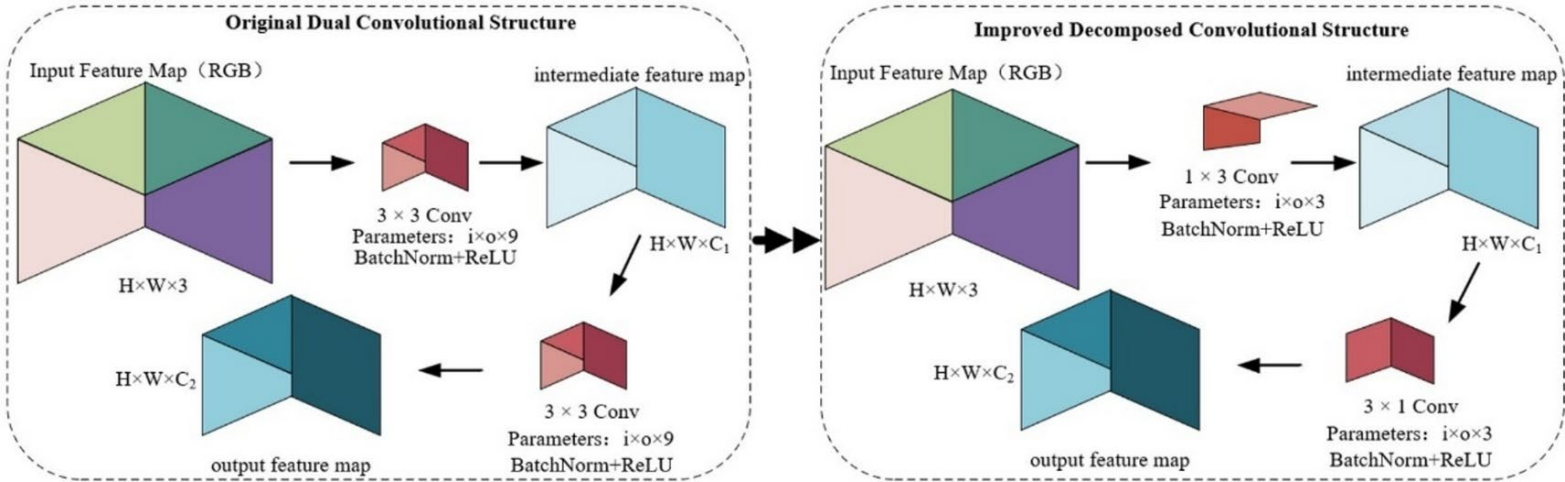
The $3\times 3$ convolution is transformed into two convolutions of $1\times 3$ and $3\times 1$.

To accelerate the model’s computation speed when processing crack images and improve work efficiency, this study modifies the original model structure, as shown in Fig. 2. The modified convolution is as follows: Double Conv originally represented the basic unit of convolution, consisting of two 3 × 3 convolutions. Instead, our model includes two convolutions of $1 \times 3$ and $3 \times 1$. Originally, $i \times o \times k$, where $i$ is the number of input channels, $o$ is the number of output channels, and $k$ is the convolution kernel, was used. We transform $i \times o \times 9$ into $2 \times i \times o \times 3$. This reduction in parameters makes the model lightweight, effectively decreasing the time required for training and inference.

### Innovative alterations

#### Feature map partitioning and multi-path propagation

A key challenge in crack detection is recognizing small cracks or detailed features. In addition, cracks may appear in images with different scales and orientations, such as fine surface cracks and larger structural cracks. The partitioning operation can effectively amplify the feature signals of these small targets, making the model more sensitive at multiple scales and thereby improving its detection accuracy for small cracks. Partitioning the feature maps can produce multiple distinct feature subspaces, with each subspace capturing different levels and types of information in the image. This partitioning helps enhance the overall expressive capability of the model, enabling it to detect different types of cracks (such as wide cracks, fine cracks, and microcracks) simultaneously. In this work, the feature maps of each layer are split into two parts along the channel dimension, as shown in Eqs. (1) and (2):

1 $$t = \left( {x_{1} \cdot x_{1} } \right) \cdot {\text{chunk}}\left( {2,1} \right)$$

In Eq. (1), $x_{1} \cdot x_{1}$ represents elementwise multiplication of the tensor $x_{1}$, whereas $chunk \left( {2, 1} \right)$ is a chunking operation that divides the tensor into multiple parts along the specified dimension.

2 $$x = {\text{concat}}\left( {x_{2} \cdot x_{2} + t\left[ 0 \right],t\left[ 1 \right],{\text{dim}} = 1} \right)$$

In Eq. (2), $x_{2} \cdot x_{2}$ represents elementwise multiplication of the tensor $x_{2}$, whereas $x_{2} x_{2} + t\left[ 0 \right]$ indicates elementwise addition of the result of $x_{2} \cdot x_{2}$ with $t\left[ 0 \right]$. The $concat$ operation signifies concatenation along the specified dimension (in this case, $dim = 1$).

Moreover, partitioning can be used to separate highly correlated features, thereby reducing the transmission of redundant information. This approach ensures that important features are not obscured by unrelated or redundant information during the fusion process, preventing information loss.

Partitioning also enables feature maps at the same layer to be processed differently. Some feature maps are further downsampled (compressed) in the encoder path, whereas another portion of the feature maps is used for fusion (skip connections) in the decoder path. This segmentation approach effectively preserves and utilizes multiscale information, enhancing the richness of feature representation.

The multipath propagation used in this study transmits and fuses feature information through multiple paths, enabling the effective combination of the global context with local detail information. This enhanced fusion improves the clarity and coherence of crack edges, increasing the accuracy of the crack detection results. Since noise is always present in images of cracks, multipath propagation employs different paths to capture various feature information from the image. Even if some paths experience information loss or noise, the model can still rely on features from other paths for discrimination. This redundant design strengthens the robustness of the network, increasing its resilience to uncertainties and variations in crack detection.

Images often contain various interferences (complex backgrounds) surrounding cracks, and multipath propagation enables the model to simultaneously process information from different contexts, allowing features at various resolutions and semantic levels to be combined. This diverse feature information helps capture various shapes, directions, and structural characteristics of cracks. By fusing low-level edge and texture information with high-level semantic information, the model can achieve a more comprehensive understanding of the morphology and location of cracks, enhancing its ability to distinguish between complex backgrounds and cracks.

#### Layer-wise fusion of multiscale features

This paper employs multiscale feature fusion during the network’s upsampling and downsampling process, as shown in Eqs. (3) and (4). This method captures crucial information in the feature map by computing the global average and generates feature weights to adjust each channel of the input feature map. This approach is similar to the attention mechanism, which enhances the expressive capability of the model. Layer-wise fusion allows the model to handle images of cracks under various conditions, such as different lighting, materials, or perspectives. Because multiscale features capture information at different scales and contexts, the model demonstrates better generalization ability in different environments and conditions, improving the detection performance on new samples.

3 $$x1 = {\upsigma }\left( {{\text{Adapt}}\left( {x1} \right)} \right)$$

4 $$x2 = {\upsigma }\left( {{\text{Adapt}}\left( {x2} \right)} \right)$$

In Eqs. (3) and (4), the input tensors ${x}_{1}$ and ${x}_{2}$ are processed through the adaptive function $Adapt$, followed by a nonlinear transformation via the activation function $\sigma$, resulting in new outputs $x_{1}$ and $x_{2}$.

After generating the weights, we perform weighted processing on the feature maps and then combine the various feature maps through concatenation. This process improves the information interaction between different features and helps retain more detailed characteristics.

This strategy of layer-wise fusion of multiscale features introduces richer contextual information through skip connections and combines features at different scales, which significantly enhances feature representation and the accuracy of the final output. This approach to fusing multiscale features improves the accuracy of the segmentation results.

Ultimately, the U-Net-FML model can accurately locate cracks at different levels. Low-level features provide detailed edge information, whereas high-level features help determine the overall structure of cracks. This fusion of layered information can increase the precision of crack localization, ensuring that the contours of the cracks are accurately segmented.

The advantages mentioned in the previous improvements will be further validated and demonstrated in the subsequent experimental results.

## Dataset

**Fig. 3:**
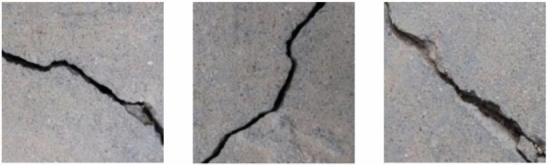
Crack-Detection-Master crack images.

**Fig. 4:**
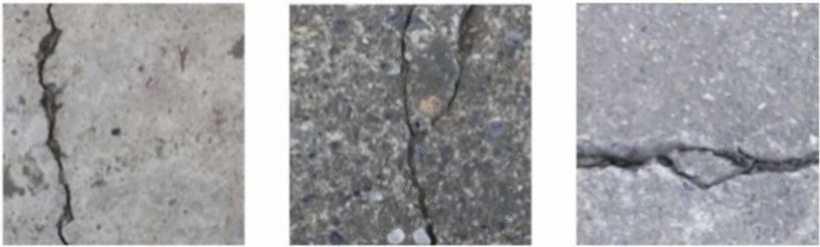
Self-collected images of cracks in natural aggregate pavements.

**Fig. 5:**
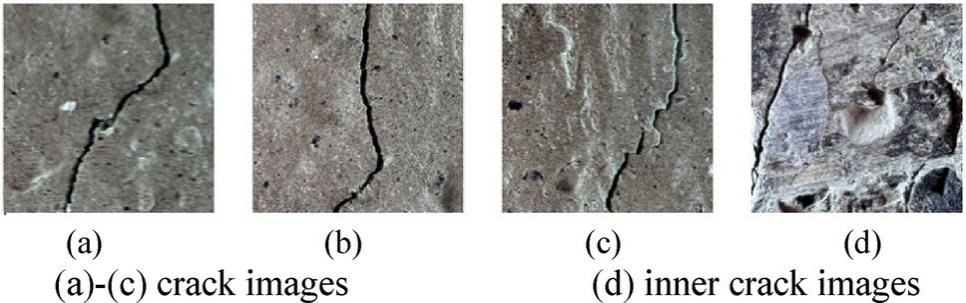
Self-collected image of cracks in the pavement of recycled steel slag.

**Fig. 6:**
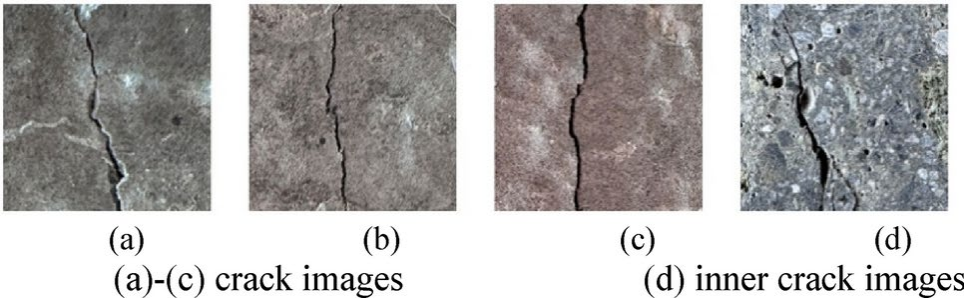
Self-collected recycled aggregate pavement crack images.

The dataset in this study consists of a public dataset and a self-collected dataset. The public dataset is obtained from the Crack-Detection-Master (CDM)^52^ crack image dataset, which contains a total of 6,077 images of cracks in concrete bridges and buildings. The self-collected dataset consists of 960 images, including 550 photos of traditional aggregate concrete pavement cracks, 200 photos of steel slag aggregate cracks, and 210 photos of recycled aggregate concrete pavement cracks. All the images in this dataset are captured with a high-resolution camera. To distinguish actual crack pixels, the self-collected photos are subjected to further pixel segmentation and converted into images with a resolution of 224 × 224. Some sample crack images are shown in Figs. 3, 4, 5 and 6. The incorporation of the Crack-Detection-Master public dataset enriches the diversity of the training data and enhances the model’s generalization ability. Additionally, this training method allows for horizontal comparisons with other research findings to assess the model’s relative performance, reduce bias, and validate the model’s universality.

**Fig. 7:**
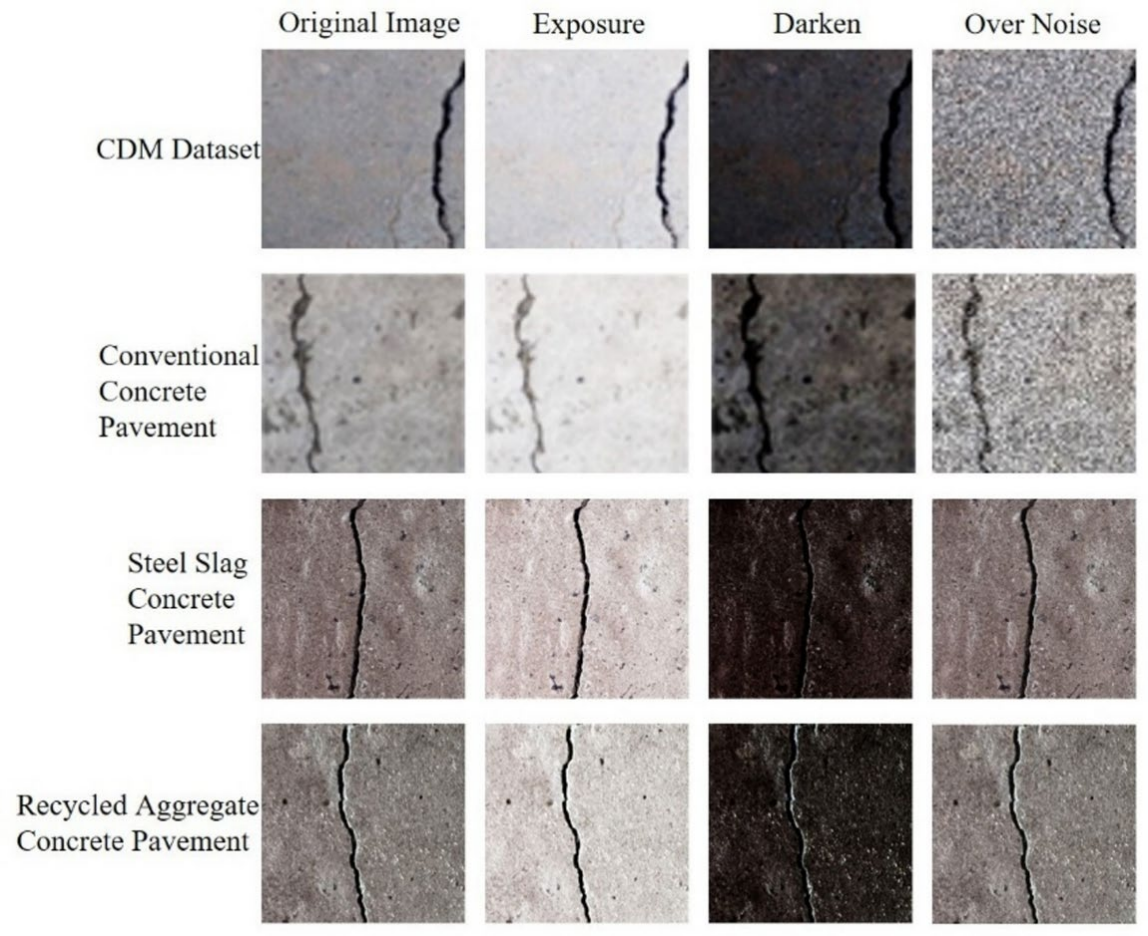
Comparison of different operations on concrete with different aggregates.

The complexity of concrete damage detection is often influenced by the variability of observed weather conditions, which affect the accuracy of the damage detection process to varying degrees. Therefore, to simulate crack images under various lighting and weather conditions and address the limited scope of the original dataset, we applied a series of image processing techniques, including exposure adjustment, darkening, and noise addition, to create a comprehensive and diverse dataset. This enhances the model’s robustness and generalization in detecting pavement cracks under different conditions, as shown in Fig. 7. The appearance of cracks in concrete pavements with different aggregates is almost indistinguishable, indicating that the model has the ability to detect cracks in concrete pavements with different aggregates.

**Fig. 8:**
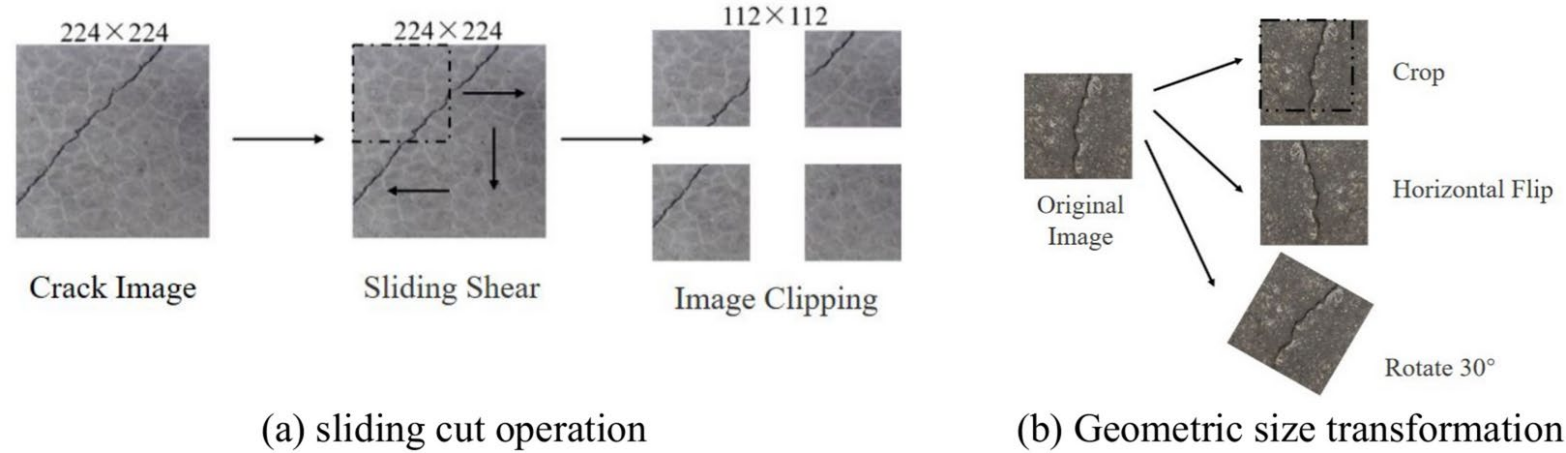
Geometric augmentation of crack images.

To prevent data leakage that could lead to overfitting during model training and inflated evaluation metrics, this study first divides the 960 acquired crack images into training, validation, and test sets at a ratio of 8:1:1, with all data augmentation operations performed exclusively on the training set. To further improve the training effectiveness of the model, sliding cut operations are applied to the images in the training, validation, and test sets. This operation not only augments the dataset but also processes large-pixel images into smaller-pixel images, allowing the model to focus more on crack pixels^51^, thereby increasing the accuracy of the model’s predictions. Additionally, to strengthen the model’s ability to recognize crack images of different angles and sizes, random cropping, flipping, and rotation are applied to the training set images, as shown in Fig. 8.

### Image pixel enhancement

**Fig. 9:**
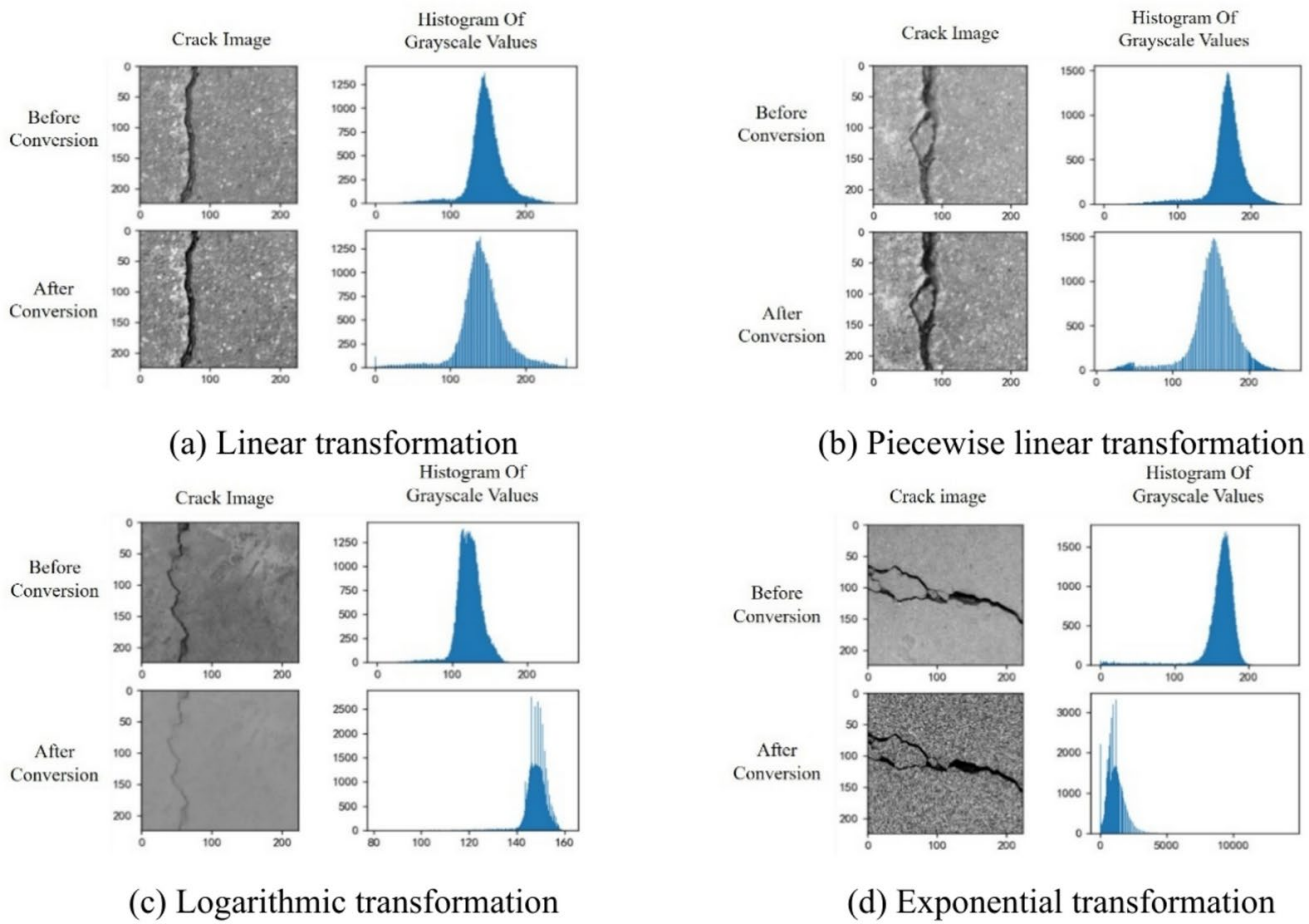
Different methods of grayscale transformation.

To maximize the optimization of the model’s training results and enhance its robustness, preprocessing steps such as image pixel enhancement need to be applied to the data. This step not only prevents overfitting of the model but also allows the model to learn more pixel features, increasing the number of training images and improving the model’s generalization ability. Data augmentation is typically carried out at both the pixel level and the geometric dimension level of the images. Pixel-level methods can be further categorized into spatial domain processing and frequency domain processing, depending on the scope of application. Spatial domain processing primarily includes methods such as gray-level transformation and histogram equalization, whereas frequency domain processing includes image smoothing techniques such as Gaussian filtering and median filtering. This model primarily employs gray-level transformation, which involves converting the original image into a grayscale image and then altering the gray values of the pixels without changing their specific locations, thereby highlighting the features of the areas of interest. The transformation methods can be classified into three types: linear transformation, piecewise linear transformation, and nonlinear transformation. Nonlinear transformation involves processing the gray values of the image using nonlinear functions. The two main nonlinear transformation methods currently in use are logarithmic transformation and exponential transformation. Figure 9 shows the four grayscale transformation methods mentioned above.

**Fig. 10:**
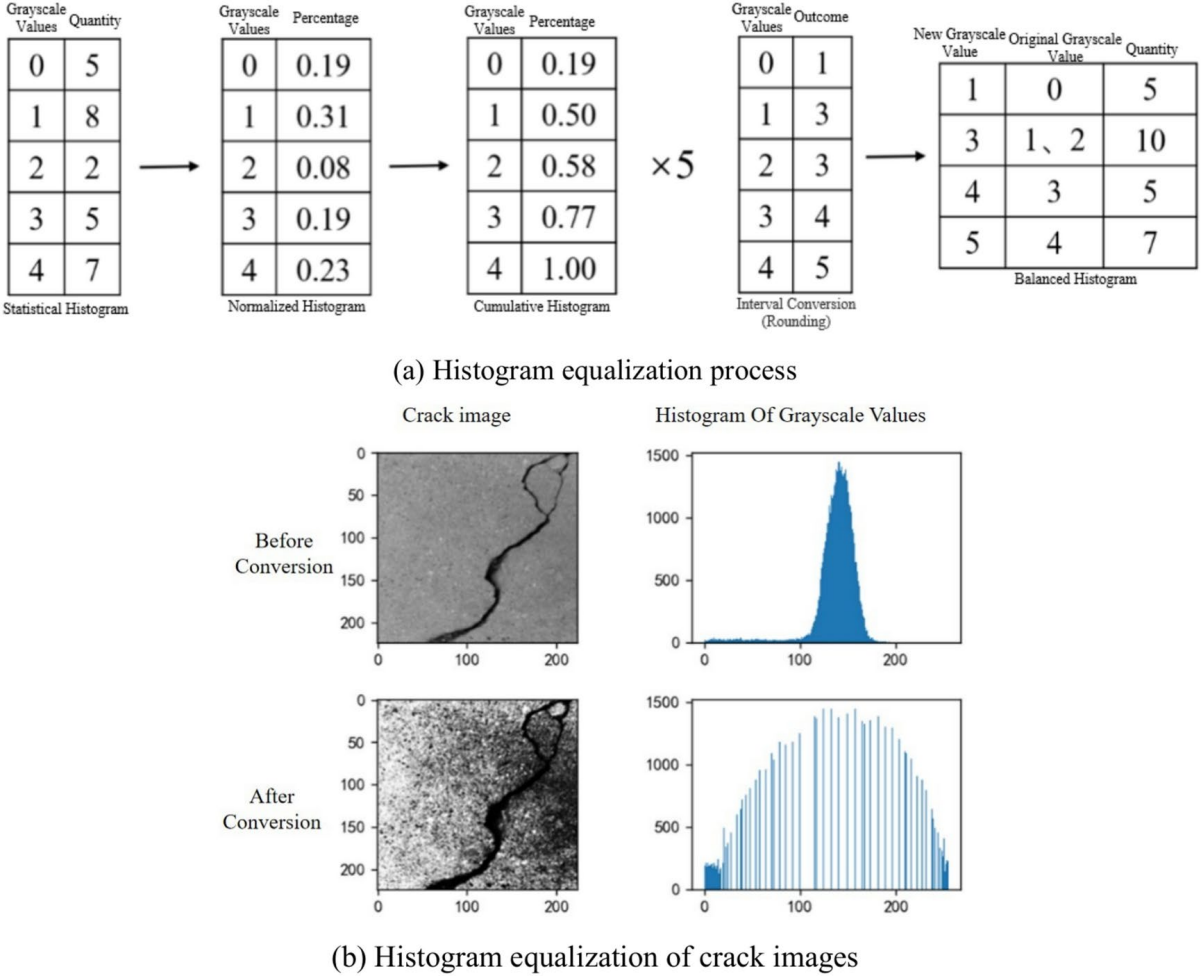
Histogram equalization of crack images.

Grayscale histogram equalization is performed using the OpenCV library in Python to adjust the pixel value distribution of the original image, making it more uniform and enhancing the image contrast. The histogram equalization process is shown in Fig. 10a. After histogram equalization, the variety of grayscale values decreases, and the distribution of grayscale values becomes more uniform. Figure 10b shows that the grayscale values of the original image are concentrated in the range of [100, 200]. After histogram equalization, the grayscale values of the image are more evenly distributed across the range of [0, 255], which diminishes the features of the crack image. Weakening the features of crack images can test the model’s performance when faced with subtle crack characteristics. If the model can still accurately detect the presence of cracks after these features have been diminished, the model possesses strong robustness and can handle various complex situations in practical applications. Moreover, this approach compels the model to learn the essential characteristics of cracks in greater depth rather than merely relying on obvious image features.

### Image smoothing processing

The purpose of image smoothing processing is to eliminate noise and smooth the image; additionally, smoothing blurs the original image to highlight the overall large features while ignoring small details. The primary methods include mean filtering, median filtering, Gaussian filtering, and bilateral filtering. In this study, Gaussian filtering and median filtering are used.

**Fig. 11:**
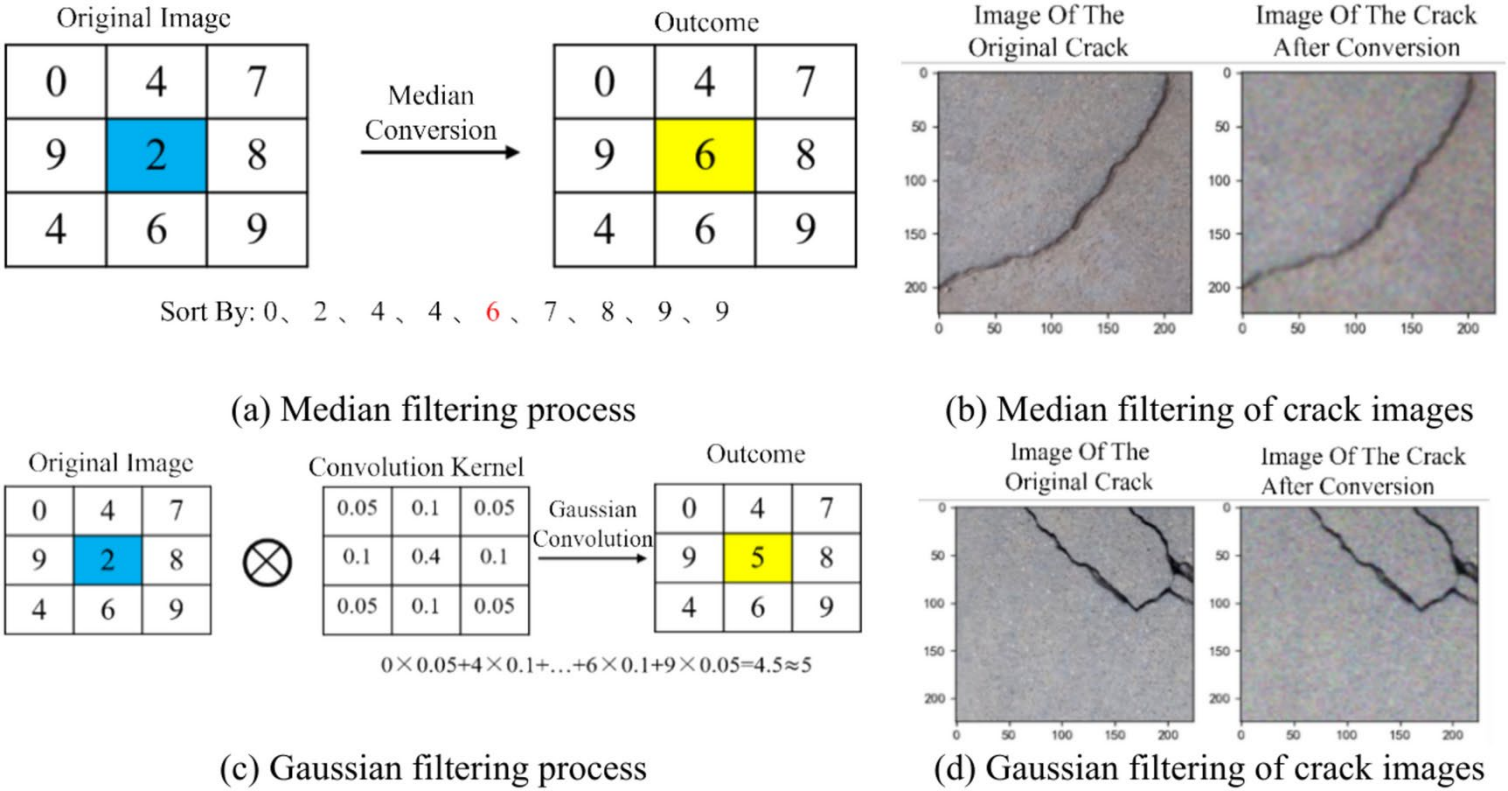
Image Smoothing Processing.

The main principle of median filtering is to replace the grayscale value of a pixel in the image with the median of its own value and the grayscale values of its neighboring pixels, as shown in Fig. 11a and b. The main principle of Gaussian filtering is to use a convolution kernel with weight parameters to perform a weighted calculation on the gray values of the pixels in the area being transformed, and the cumulative result of this calculation serves as the transformed gray value for that pixel, as shown in Fig. 11c and d. After median filtering, the image becomes blurry, losing many detail pixel points and only roughly highlighting the pixel features of the cracks. After Gaussian filtering, the image is also blurred, with results similar to those of median filtering.

Image smoothing processing removes potential random noise points from the crack image, resulting in a cleaner appearance. Smoothing generally involves averaging or applying weighted averages to the pixel values in the image, resulting in a more gradual transition of pixel values at the edges. Although the blurring of edges may somewhat affect the observation of crack details, smoothing can also reduce interference from false edges, making it easier for subsequent algorithms to extract the true edges of cracks accurately.

## Experimental results and discussion

### Implementation details

**Fig. 12:**
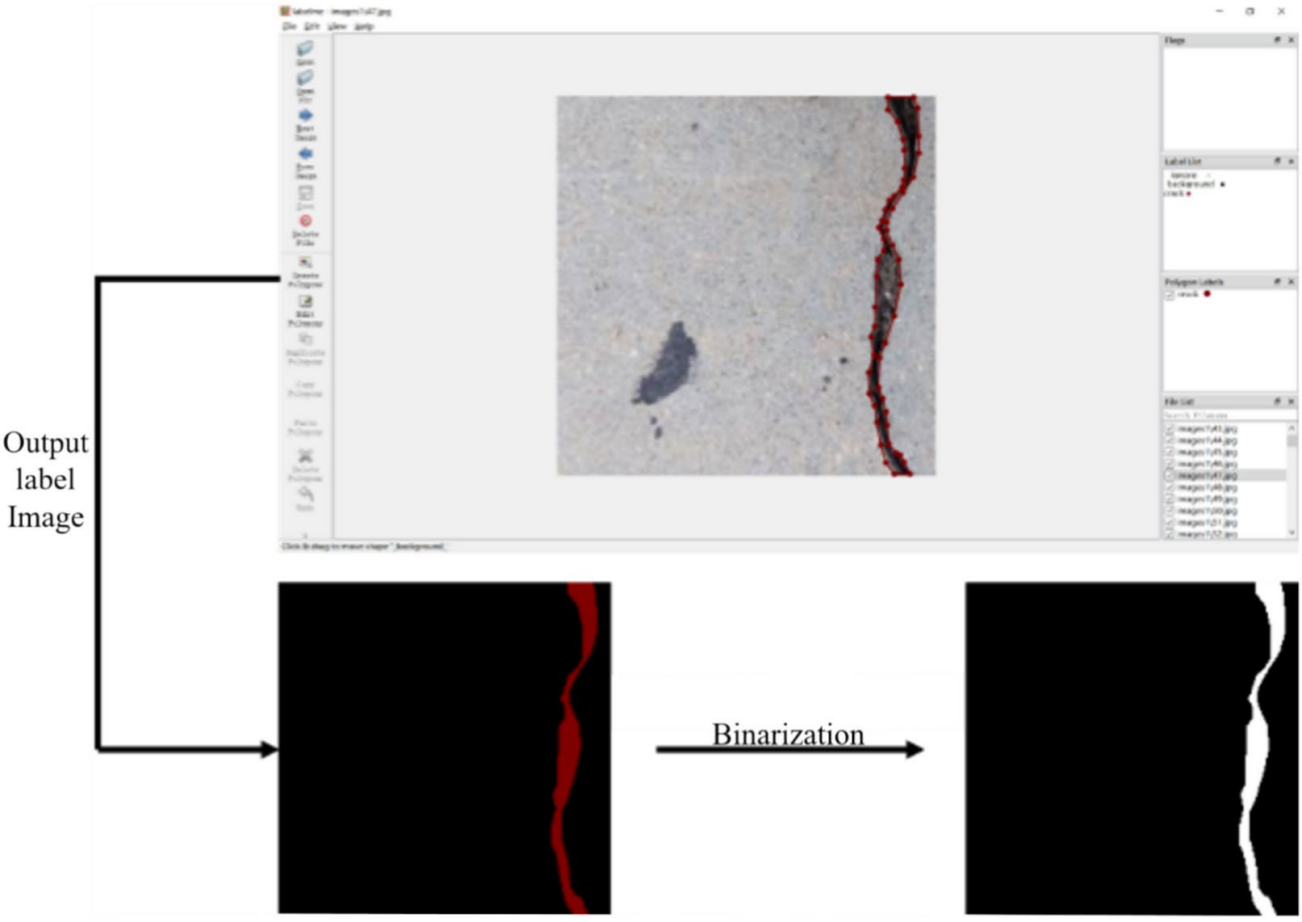
Labeling crack images using Labelme software.

All the experiments are conducted using the PyTorch framework on the CentOS 7 operating system implemented on a workstation utilizing the GPU mode of the Linux system. Compared with central processing units (CPUs), GPUs can perform deep learning tasks more quickly and efficiently because of their high parallel processing capabilities. The two GPUs employed are NVIDIA Ampere A100 units, each with 80 GB of memory. In this study, a supervised neural network model is used for training. In supervised training, the model not only receives training images as input but also requires corresponding labeled images to evaluate the training results. The network optimizes its parameters on the basis of this evaluation, aiming to make the predicted results closer to the labeled images. Labeled images corresponding to the 7,037 collected concrete crack images are created with Labelme software for manual annotation, as shown in the Fig. 12. First, the label tags are set as _ignore_, _background_, and _crack_, which correspond to ignorable areas, black, and white, respectively, in the binary labeled image. Using the region selection tool in the system, the crack areas are extracted as anchor points, generating the corresponding JSON files. After running the process, the annotated crack pixels in the image are automatically segmented, resulting in a labeled image that expresses the foreground and background using only single pixel types. The software directly outputs the foreground of the image in red [255, 0, 0], which needs to be binarized to convert the red foreground into white, as shown in Fig. 12.

### Evaluation criteria

To evaluate the model’s performance for crack detection, this study introduces four key evaluation metrics: precision (Pr), recall (Re), F_1_ score (F_1_), and mean intersection over union (MIoU). These metrics are defined by Eqs. (5–8). Precision is the ratio of true positives to the total positive samples, whereas recall is the proportion of positive results that are correctly predicted as positives. The F_1_ score is the harmonic mean of precision and recall, providing a comprehensive assessment of model performance. The MIoU metric measures segmentation accuracy in semantic segmentation and is calculated as the average ratio of the intersection to the union of the predicted segmentation area and the true segmentation area. In the crack segmentation task, crack pixels are classified as positive samples, and non-crack pixels are classified as negative samples. On the basis of the actual conditions of the concrete pavement and the prediction results, the pixels can be categorized into four scenarios, as shown in Table 1.

**Table 1:** All the results of the predicted case and the ground truth case.

Ground Truth	Predicted	
	Crack	Non-crack
Crack	True positive (TP)	False negative (FN)
Non-crack	False positive (FP)	True negative (TN)

Then, Precision, Recall, F_1_, and MIoU could be defined as:

5 $${\text{Precision}} = \frac{TP}{{TP + FP}}$$

6 $${\text{Recall}} = \frac{TP}{{TP + FN}}$$

7 $$F_{1} = {2} \times \frac{{{\text{Precision}} \times {\text{Recall}}}}{{{\text{Precision}} + {\text{Recall}}}}$$

8 $${\text{MIoU}} = \frac{GroundTruth \cap Prediction}{{GroundTruth \cup Prediction}}$$

TP, FP, TN, and FN represent true positives, false positives, true negatives, and false negatives, respectively. The F_1_ score and MIoU values are two of the primary indicators utilized in this paper to assess the performance of the trained network.

### Ablation experiments

To evaluate the impact of the proposed innovations on model performance and understand the contribution of each component, we present optimization tests that focus on different elements of the model. The U-Net-FML network incorporates these innovations to improve small crack detection accuracy. As shown in Table 2, we designed three optimization scenarios, progressively adding feature map partitioning, multi-path propagation, and layer-wise multi-scale feature fusion to the U-Net architecture to assess the effect of each module on performance.

**Table 2:** Comparative study results of different models.

Model	Evaluation Metrics			
	MIoU	F_1_ score	Precision	Recall
U-Net-F	72.1	69.3	78.1	62.4
U-Net-FM	75.3	72.8	82.2	65.2
U-Net-M	73.5	70.3	79.3	63.2
U-Net-ML	76.1	73.9	83.2	66.2
U-Net-L	74.8	71.4	80.2	64.1
U-Net-FML	76.4	74.2	84.2	66.4

U-Net-F integrates feature map partitioning, improving efficiency in processing large images and enhancing the capture of local features. While this boosts efficiency, the F_1_ score slightly decreases to 69.3. U-Net-FM combines feature map partitioning with multi-path propagation, improving local feature learning and information flow. This results in a notable increase in Precision (82.2) and F_1_ score (72.8), though Recall drops slightly to 65.2. U-Net-M emphasizes multi-path propagation, improving information flow and handling diverse scenarios more effectively. It boosts both F_1_ score (70.3) and Recall (63.2), but it slightly limits the ability to capture fine details. U-Net-ML combines multi-path propagation with layer-wise multi-scale feature fusion, significantly enhancing the model’s multi-scale learning. This results in the highest F_1_ score (73.9) and MIoU (76.1), though at the cost of increased training time. U-Net-L focuses on layer-wise multi-scale feature fusion, improving the model’s ability to perceive targets at various scales. This results in a higher MIoU (74.8) and Recall (64.1), making it particularly effective for multi-scale tasks.

Finally, U-Net-FML combines all three modules (F, M, and L), addressing local, global, and multi-scale features. It achieves the best performance across all metrics: MIoU (76.4), Precision (84.2), Recall (66.4), and F_1_ score (74.1). These results demonstrate the complementary effects of the innovations, highlighting the superior segmentation performance of U-Net-FML.

### Comparative experiments

This paper introduces the U-Net-FML model, an enhanced deep learning framework based on U-Net, which employs feature map partitioning, multipath propagation, and layer-wise fusion of multiscale features to achieve pixel-level crack segmentation. During training, the performance of this improved detection method is compared against those of traditional semantic segmentation models, including U-Net, U-Net++^53^, DeepLabv3+^54^, DANet^55^, UperNet^56^, and TransNet^57^. U-Net, U-Net++, and DeepLab v3+share a symmetric encoder-decoder structure, with U-Net++building upon U-Net by introducing dense skip connections and multiple sub-encoder-decoder pathways, whereas DeepLab v3+incorporates an atrous spatial pyramid pooling (ASPP) module in the encoder. DANet uses a dual-attention mechanism with parallel attention modules, UperNet employs a feature pyramid network (FPN) as its backbone with a pyramid pooling module (PPM) to capture the global scene context, and TransNet integrates a transformer module with self-attention for enhanced feature extraction. Each of these models demonstrates strong performance in tasks demanding precise boundary detection and handling complex backgrounds, providing a benchmark for comparison with the proposed U-Net-FML network in this study.

To ensure a fair comparison, all the models are trained under consistent experimental conditions and with identical network parameters until each model reaches convergence for optimal performance. This approach allows for a straightforward determination of the best-performing model. Table 3 shows the detection results of the seven models trained on the same test set. The U-Net-FML model achieves the highest MIoU, F_1_ score, precision, and recall values, indicating superior performance and the best recognition effectiveness among all the models.

**Table 3:** Comparative study of results from different modules.

Model	MIoU	F_1_ score	Precision	Recall
U-Net-FML	**76.4**	**74.2**	**84.2**	**66.4**
U-Net	74.3	71.2	81.2	63.2
U-Net++	75.5	73.2	83.3	65.2
Deeplab v3+	74.3	73.3	82.2	66.2
DANet	73.8	70.9	79.2	64.1
UperNet	73.5	69.8	82.2	60.7
TransNet	75.9	73.6	**84.2**	65.3

The bolded values represent the best results achieved by the seven network models across four evaluation metrics (MIoU, F_1_ score, Precision, Recall) under identical training conditions and datasets. The U-Net-FML network stood out with exceptional performance in key metrics: it achieved an MIoU of 76.4, an F_1_ score of 74.2, and a Recall of 66.4, all significantly surpassing the other models. In terms of Precision, the U-Net-FML network performed on par with TransNet, both achieving a top value of 84.2. These bolded values highlight the U-Net-FML network’s superior overall performance in this experiment.

**Fig. 13:**
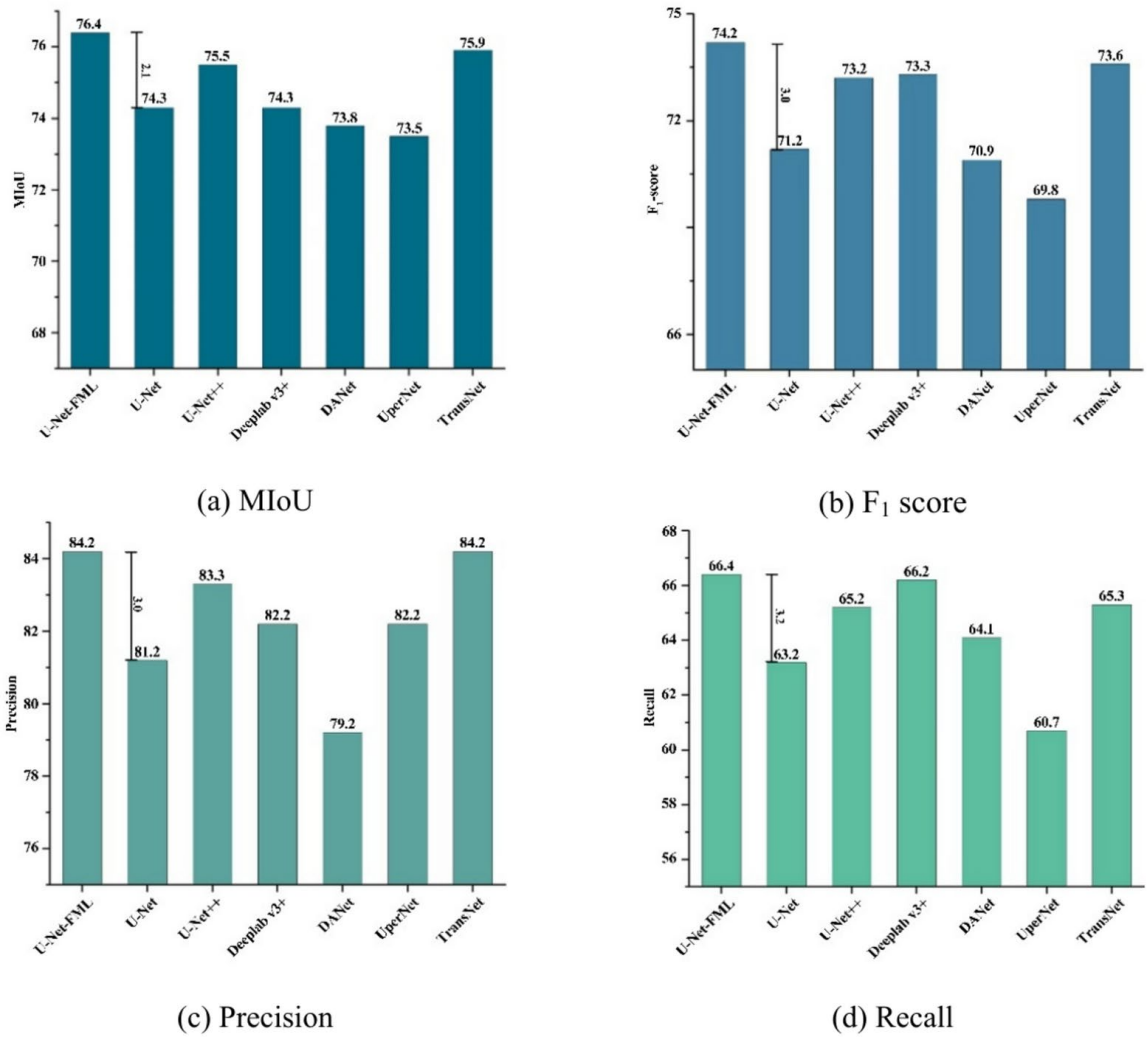
(**a**–**d**) Comparison of MIoU, F_1_ score, Precision, and Recall among the seven models.

To demonstrate the superior performance of the U-Net-FML model, we compare its MIoU, F_1_ score, precision, and recall metrics across different models, as shown in Fig. 13. According to Fig. 13 and Table 3, under consistent operating conditions and network parameters, U-Net-FML performs exceptionally well, with MIoU, F_1_ score, precision, and recall values of 76.4%, 74.2%, 84.2%, and 66.4%, respectively, significantly outperforming the other models.

MIoU is a critical metric for evaluating pixel-level accuracy, and the proposed model achieves the highest MIoU score among all compared models, outperforming them by 2.1%, 0.9%, 2.1%, 2.6%, 2.9%, and 0.5%, respectively, indicating significantly greater detection accuracy than that of other semantic segmentation networks. The F_1_ score, which balances precision and recall, is also the highest for the proposed model, surpassing UperNet by 4.4% and DANet by 3.3%, demonstrating the accuracy of the proposed model in identifying cracks. In terms of precision, an indicator of prediction accuracy, the proposed model scores 5.0% higher than DANet and 3.0% higher than U-Net does, suggesting fewer false positives. Finally, the recall, which measures the model’s ability to detect the quantity of cracks and assess missed detections, shows that the proposed model outperforms UperNet by 5.7% and U-Net by 3.2%, indicating its ability to capture the majority of crack information in images with high detection accuracy. These four key metrics—MIoU, F_1_ score, precision, and recall—are comprehensive indicators of pixel-level detection accuracy, revealing that the U-Net-FML model achieves the greatest robustness, precision, and generalization capabilities among all the models in the comparison. This result demonstrates the effectiveness of progressively applying strategies such as feature map partitioning, multipath propagation, and multiscale feature fusion, substantially enhancing the overall performance of the proposed model.

To clearly highlight the efficiency and performance advantages of the proposed U-Net-FML model, we summarize the parameter counts and Gflops (Giga Floating Point Operations Per Second) of the experimental models in Table 4. The U-Net-FML model achieves the lowest parameter count, with only 7.68 million parameters, compared to 7.76 million for the standard U-Net, 9.02 million for U-Net++, 40.80 million for Deeplab v3+, 58.61 million for DANet, 60.27 million for UperNet, and 40.12 million for TransNet. This reduction is attributed to optimizing the convolution operations by replacing the original 3 × 3 convolutions with a combination of 1 × 3 and 3 × 1 convolutions. This change not only reduces the computational load but also decreases the total parameter count.

**Table 4:** Comparison of parameters between the improved and traditional segmentation networks.

Model	Parameters(M)	Gflops(G)
U-Net-FML	7.68	43.67
U-Net	7.76	41.92
U-Net++	9.02	51.15
Deeplab v3+	40.80	25.12
DANet	58.61	50.61
UperNet	60.27	60.13
TransNet	40.12	35.79

In terms of computational complexity, the U-Net-FML model operates at 43.67 Gflops, which, though higher than some models, is still lower than U-Net++ (51.15 Gflops), DANet (50.61 Gflops), and UperNet (60.13 Gflops). Despite this, our U-Net-FML model outperforms these models in key metrics such as MIoU, Recall, Precision, and F1 score. This demonstrates that our model achieves superior segmentation accuracy while maintaining lower computational overhead and reduced memory usage.

The lightweight and efficient design of the U-Net-FML model makes it highly suitable for environments with limited computational resources, without compromising performance. These benefits emphasize the practical value of our model, as it offers higher accuracy with fewer parameters and lower computational costs, making it an ideal solution for real-world applications where both efficiency and performance are critical.

**Fig. 14:**
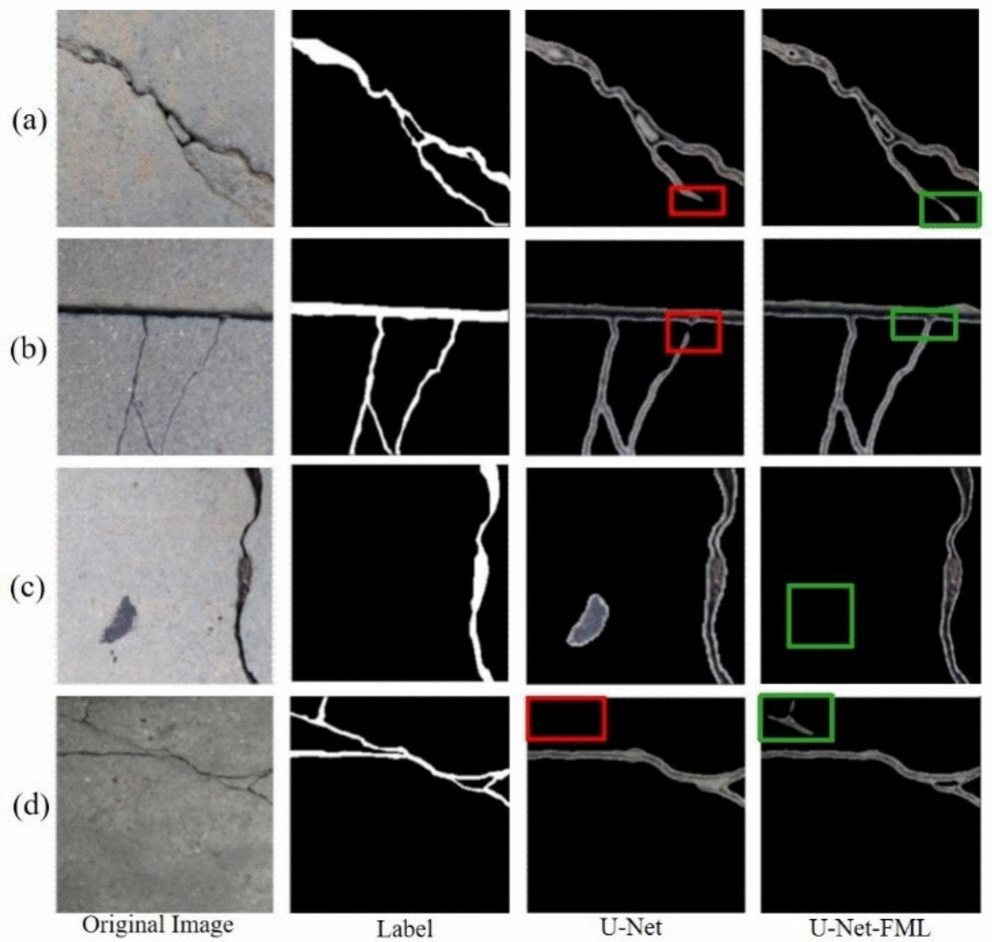
Comparison of crack image recognition prediction results from experimental models.

Figure 14 shows the detection results of U-Net and U-Net-FML on two representative crack images. The first column shows the original crack images, the second column presents the corresponding labeled images, and the last two columns display the predictions from each algorithm. Additionally, to simulate the complex nature of real-world pavement cracks, test images from the dataset were randomly selected during the training process.

The results in Fig. 14 demonstrate that both algorithms are able to detect the basic outlines of cracks, but there are differences in their handling of details. The U-Net model produces both false positives and missed detections, particularly when dealing with complex, small, or irregularly shaped cracks, leading to insufficient continuity in detection. In these cases, additional postprocessing may be required for high-precision crack identification. In contrast, U-Net-FML reduces redundant information transfer through operations such as feature map partitioning, multipath propagation, and layer-wise fusion of multiscale features. This approach prevents information loss and results in a more accurate representation of the crack distribution, demonstrating superior detection performance compared with the traditional U-Net model. As clearly shown in the third and fourth rows, U-Net-FML is able to accurately identify cracks that U-Net fails to detect, making the improved model more suitable for concrete crack detection and validating the effectiveness of the proposed enhancement.

The primary reasons for the differences in segmentation accuracy between U-Net and the model proposed in this study are twofold. First, the multiscale feature fusion in our model improves the model’s ability to capture both fine details and global information, which increases its capacity to detect cracks of varying sizes and complexities. Second, the feature map partitioning and multipath propagation techniques reduce the transmission of redundant information and prevent the loss of crucial details. In contrast, U-Net lacks these mechanisms, which may cause difficulties in processing fine details or recognizing complex features, particularly when dealing with small or irregular cracks, leading to reduced accuracy. Overall, compared with U-Net, the proposed U-Net-FML model provides a more efficient approach for crack detection, with a better understanding of global information.

**Fig. 15:**
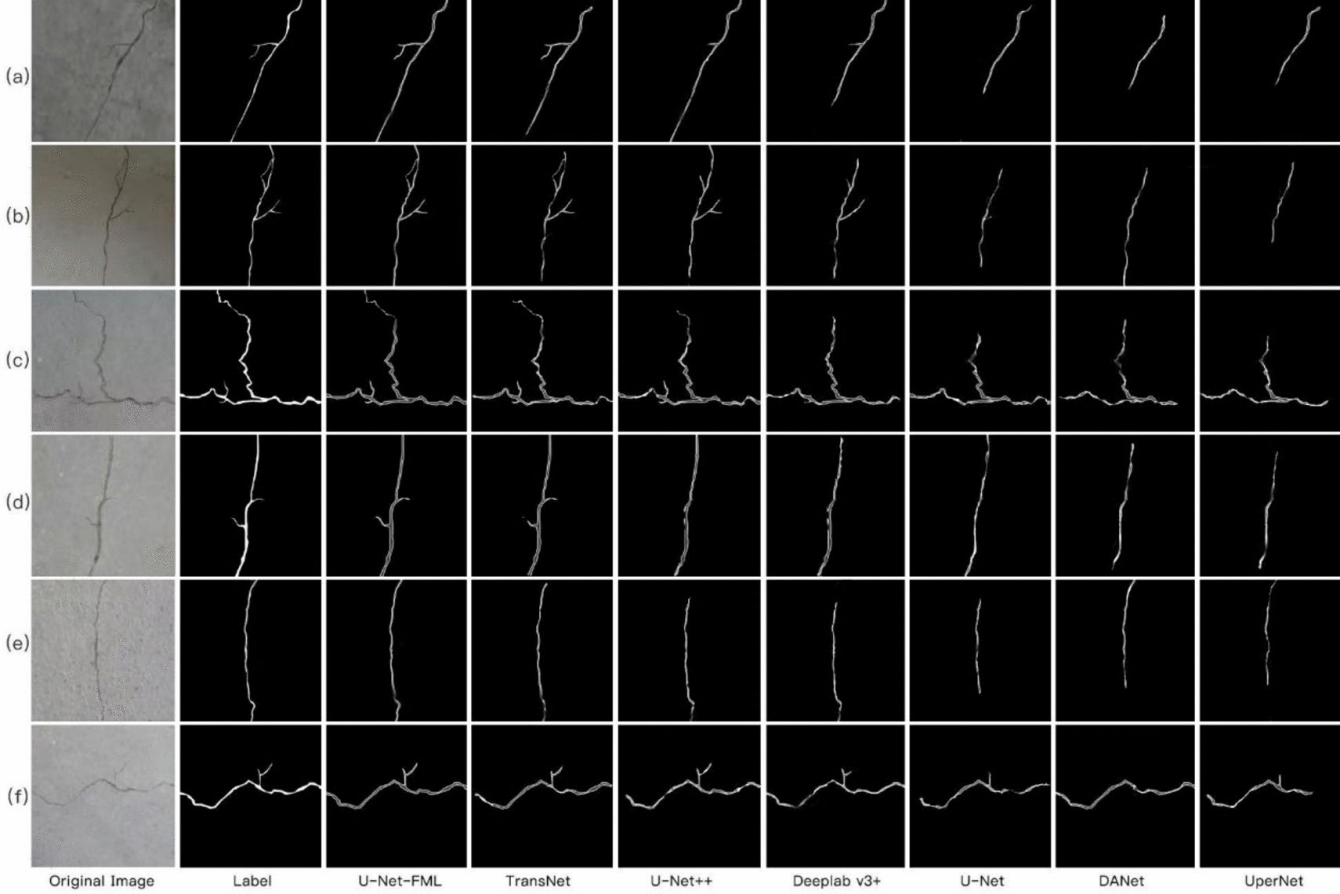
Comparison of experimental models’ recognition and prediction results on small crack images.

Figure 15 presents the visualization results for the small crack dataset, which includes six pavement crack images, their corresponding ground truth labels, and the predicted results generated by various models. The first and second columns display the original images and ground truth labels, respectively. The subsequent columns (from the third to the eighth) show the predicted crack regions produced by the U-Net-FML, TransNet, U-Net++ , DeepLabv3+, U-Net, DANet, and UperNet models. All test images are randomly selected to reflect the crack prediction performance of the seven models on small cracks.

As seen in Fig. 15, the U-Net-FML model outperforms the others in small crack segmentation. It excels at capturing fine details and edge information, leading to more accurate delineation of crack regions. In comparison to traditional models like U-Net and U-Net++ , U-Net-FML demonstrates superior accuracy and robustness in segmenting small cracks. While models such as DeepLabv3+, DANet, UperNet, and TransNet perform well in some cases, they still show significant errors, particularly with small cracks in complex backgrounds.

These results emphasize the strong performance of the U-Net-FML model in detecting small cracks, even under challenging and varied conditions. Its ability to maintain high accuracy and robustness across different scenarios highlights its broad applicability and adaptability to various crack types.

## Conclusion

Considerable progress has been made in pavement crack identification, particularly in classification, detection, and segmentation tasks. With advances in deep learning, convolutional neural network (CNN)-based models, such as YOLOv5 and CrackNet, have shown promising results in crack detection. However, despite the improvements in detection accuracy and efficiency, pixel-level crack detection still faces challenges, particularly in cases with blurry crack edges or complex backgrounds. Therefore, achieving higher accuracy in crack detection while maintaining speed and robustness remains a critical challenge. To address this, we propose a high-precision U-Net-based semantic segmentation model, U-Net-FML, which aims to enhance pixel-level crack recognition accuracy and efficiency by optimizing network structure and feature extraction. During the training phase, the effectiveness of the U-Net-FML model is validated through comparative experiments with seven different networks. The results show that U-Net-FML outperforms other traditional semantic segmentation networks. Specifically, the model achieves MIoU, F_1_ score, precision, and recall values of 76.4%, 74.2%, 84.2%, and 66.4%, respectively. The MIoU, an important metric for evaluating semantic segmentation accuracy, provides a clear measure of pixel-level precision. Compared with the other models, U-Net-FML improved the MIoU by 2.1%, 0.9%, 2.1%, 2.6%, 2.9%, and 0.5%, demonstrating superior precision and faster performance when handling complex images.

Therefore, the following conclusions are derived:

1. This study reduces the number of parameters in the training process by modifying convolutional operations, making the proposed U-Net-FML model more lightweight and efficient. This adjustment addresses the long training times and high computational demands of the original U-Net model, enabling faster deployment and real-time processing of large-scale pavement crack detection, which is critical for road maintenance.
2. By utilizing feature map partitioning and multipath propagation, the model’s ability to distinguish cracks from complex backgrounds has been significantly enhanced. This approach not only improves crack detection in various environmental conditions but also ensures robustness in real-world pavement inspection scenarios where cracks often blend with the surrounding texture and noise. The model’s ability to generalize across these factors enhances its suitability for practical road maintenance tasks.
3. The U-Net-FML model integrates multiscale feature fusion, layer-wise processing, and image enhancement to effectively capture both contextual information and fine details. This improves crack detection accuracy, even under challenging conditions such as varying lighting, weather, and road surfaces. By utilizing a dataset that reflects real-world complexities, the model enhances its robustness and efficiency, making it well-suited for infrastructure monitoring and maintenance.

## Data Availability

Data sets generated during the current study are available from the corresponding author on reasonable request.
